# The Role of Repetitive Transcranial Magnetic Stimulation in Treating Alcohol Use Disorder: Neural Mechanisms, Efficacy, and Future Directions

**DOI:** 10.31083/AP49374

**Published:** 2025-12-23

**Authors:** Guannan Li, Yinghuan Cao, Jun Zhong

**Affiliations:** ^1^Department of General Psychiatry, Guangzhou Civil Affairs Bureau Psychiatric Hospital, 510430 Guangzhou, Guangdong, China; ^2^Department of General Psychiatry, Guangzhou Kangning Hospital, 510430 Guangzhou, Guangdong, China; ^3^Department of Pharmacy, Guangdong Provincial Hospital of Traditional Chinese Medicine, The Second Affiliated Hospital of Guangzhou University of Traditional Chinese Medicine, 510120 Guangzhou, Guangdong, China; ^4^Department of Addiction Psychiatry, The Affiliated Brain Hospital, Guangzhou Medical University, 510370 Guangzhou, Guangdong, China; ^5^Key Laboratory of Neurogenetics and Channelopathies of Guangdong Province and the Ministry of Education of China, Guangzhou Medical University, 510180 Guangzhou, Guangdong, China

**Keywords:** Alcohol Use Disorder (AUD), repetitive transcranial magnetic stimulation (rTMS), medial prefrontal cortex (mPFC), dorsolateral prefrontal cortex (DLPFC), cognitive control

## Abstract

Alcohol Use Disorder (AUD) is a global health challenge, affecting 10–15% of the population, with significant social, health, and economic consequences. Although pharmacotherapies such as disulfiram, naltrexone, and acamprosate are available, their effectiveness is limited and patient adherence is often poor. Repetitive transcranial magnetic stimulation (rTMS), a non-invasive neuromodulation technique that targets neural circuits implicated in addiction. Emerging evidence suggests that rTMS may reduce alcohol craving and consumption, although results have been mixed. This review examines the neural mechanisms by which rTMS may influence AUD, summarizes current clinical evidence of its efficacy, and discusses future directions.

## Main Points

1. Repetitive transcranial magnetic stimulation (rTMS) offers a promising non-invasive intervention for Alcohol Use Disorder (AUD), with the potential to reduce alcohol cravings and enhance executive control by targeting key brain regions such as the dorsolateral prefrontal cortex (DLPFC) and medial prefrontal cortex (mPFC).

2. This review highlights a critical distinction between single-session mechanistic studies and multi-session randomized controlled trials (RCTs), demonstrating that only repeated rTMS sessions consistently lead to meaningful clinical improvements.

3. Neuroimaging evidence reveals that rTMS modulates activity and connectivity in frontal-striatal and limbic circuits, providing mechanistic insight into its therapeutic action in AUD.

4. Compared to previous reviews, this study emphasizes three under-discussed aspects: (i) the role of neuroimaging in understanding rTMS effects, (ii) the clinical relevance of differentiating study types, and (iii) personalized treatment strategies based on neural biomarkers.

5. Future directions include optimizing stimulation protocols, integrating rTMS with behavioral or pharmacological treatments, and identifying predictors of treatment response to enhance efficacy and long-term outcomes.

## 1. Introduction

Alcohol Use Disorder (AUD) is a significant global health challenge, affecting an estimated 10–15% of the global population [1]. Characterized by compulsive alcohol consumption, loss of control over intake, and a high relapse rate, AUD is associated with social, occupational, and health-related consequences. Globally, excessive alcohol use contributes to approximately 5% of the overall disease burden [2]. In China, the situation is particularly concerning, with average annual alcohol consumption among individuals aged 15 years and older reaching 8.1 liters—substantially higher than the global average of 5.8 liters [3]. Epidemiological data estimate that the one-year prevalence rate of AUD in China is 1.8%, and the lifetime prevalence is 4.4%, affecting over 20 million individuals nationwide [4]. Despite its impact, only about 25% of individuals with alcohol dependence seek treatment, and of those, nearly half relapse within one year. Relapse rates rise to as high as 70% within three years [5, 6, 7].

Existing pharmacological treatments for AUD, including disulfiram, naltrexone, and acamprosate, aim to curb alcohol intake and prevent relapse [2, 8, 9]. However, these agents often show limited long-term efficacy, low patient adherence, and are associated with adverse effects. This has prompted a growing interest in alternative, more tolerable therapeutic approaches such as repetitive transcranial magnetic stimulation (rTMS). rTMS is a non-invasive brain stimulation technique capable of modifying dysfunctional brain networks implicated in AUD [10]. Capable of modifying dysfunctional brain networks implicated in AUD. Unlike electroconvulsive therapy (ECT) and deep brain stimulation (DBS), both of which are invasive and require anesthesia or surgical implantation, rTMS is a well tolerated and associated with a more favorable safety profile [11, 12]. By delivering repetitive magnetic pulses to targeted brain regions, rTMS modulates cortical excitability, induces neuroplastic changes, and may restore functional balance within networks regulating craving and executive control [13, 14].

This review critically evaluates rTMS as a potential treatment for AUD, with a focusing on its underlying neural mechanisms, clinical efficacy, and future directions for protocol optimization. Notably, it differentiates between single-session mechanistic studies and multi-session randomized controlled trials (RCTs), thereby clarifying the temporal and methodological scope of rTMS research. Special emphasis is placed on neuroimaging studies that elucidate circuit-level changes following stimulation. Through this dual lens, the review aims to provide a nuanced and translational overview of rTMS’s role in AUD treatment.

## 2. Neural Mechanisms of rTMS in Alcohol Use Disorder

The pathophysiology of AUD is closely linked to dysfunctions within specific neural circuits, particularly those involving the prefrontal cortex and striatum. These circuits play key roles in regulating cognitive control, decision-making, and reward processing—domains that are notably impaired in individuals with AUD. Of particular interest are the medial prefrontal cortex (mPFC) and dorsolateral prefrontal cortex (DLPFC), which have been consistently implicated in the regulation of alcohol consumption and cravings [15, 16, 17, 18]. Structural and functional abnormalities in these regions, along with disruptions in the ventral and dorsal striatum, are thought to underlie the compulsive nature of alcohol use and the difficulty AUD patients experience in resisting alcohol-related cues [15, 16, 17, 18].

The mPFC is involved in reward processing and craving regulation, whereas the DLPFC is pivotal for executive function, including inhibitory control and decision-making [19, 20, 21, 22]. In those with AUD, dysregulation within these circuits manifests as impaired cognitive control and an overactive reward system, leading to a heightened sensitivity to alcohol-related cues and an inability to suppress drinking behaviors [23]. Chronic alcohol use further exacerbates these dysfunctions, creating a pathological imbalance between cognitive control and reward systems within the fronto-striatal circuitry [24].

rTMS is thought to exerts its therapeutic effects by modulating cortical excitability and promoting neuroplasticity in these affected brain regions. Mechanistically, rTMS is known to induce long-term potentiation (LTP) and long-term depression (LTD) at synaptic connections, facilitating adaptive changes in neuronal activity and connectivity [25, 26]. Stimulation of the DLPFC, for example, may enhance executive control by improving inhibitory function and decision-making processes [27]. Additionally, rTMS targeting the mPFC has been shown to attenuate the hyperactivity of the mPFC-ventral striatum (VS) circuit—a key neural substrate for craving regulation—thereby potentially decreasing alcohol cravings and enhancing relapse resistance [28].

Moreover, rTMS has been found to influence key neurotransmitter systems, including dopamine and glutamate, which play central roles in reward processing and cognitive control [29, 30]. Dysregulation of dopaminergic pathways, particularly within the mesolimbic system, is a hallmark feature of AUD, reinforcing addictive behaviors and perpetuating alcohol misuse [31]. By modulating dopaminergic Signaling within the striatum, rTMS may help attenuate reward sensitivity to alcohol. Additionally, it may bolster glutamatergic transmission in the prefrontal cortex, supporting executive function and behavioral control in individuals with AUD [32, 33].

## 3. rTMS and Alcohol Craving

Craving represents a central feature of AUD and serves as a robust predictor of relapse. The intensity of craving frequently correlates with both the likelihood and rapidity of relapse, underscoring its importance as a therapeutic target in AUD management [34, 35, 36]. Cravings are often provoked by exposure to alcohol-related cues, which activate brain regions associated with reward and affective processing, notably the ventral striatum (VS) and medial and mPFC [20]. Previous neuroimaging study has consistently demonstrated heightened activation in these areas in individuals with AUD, contributing to compulsive alcohol consumption and difficulty maintaining long-term abstinence [37].

The efficacy of rTMS to alleviating alcohol cravings has been explored through both single-session mechanistic studies and multi-session randomized controlled trials (RCTs), which differ markedly in aims and clinical relevance. Single-session studies assess short-term neural modulation, whereas multi-session RCTs evaluate cumulative therapeutic outcomes. Several multi-session RCTs have reported promising effects. For instance, Mishra *et al*. [10] applied 10 sessions of high-frequency (10 Hz) rTMS over the right DLPFC (1000 pulses/session) in a sample of 30 active and 15 sham patients, observing significant reductions in craving scores compared to sham—a clear therapeutic signal [10]. In contrast, Höppner *et al*. [38], in a trial using 20 Hz stimulation over the left DLPFC (10 sessions, 1000 pulses/session), observed no significant craving reduction, suggesting laterality and stimulation site may affect efficacy. Other short-duration or low-intensity multi-session trials have also reported null results. For example, Herremans *et al*. [39, 40] applied just one or two sessions of 20 Hz rTMS over the right DLPFC (1560 pulses/session) and found no differences in craving compared to sham, underscoring the possible inadequacy of abbreviated protocols.

In another study, Ceccanti *et al*. [41], employed a 10-session protocol (20 Hz, 1000 pulses/session) targeting the bilateral medial prefrontal cortex (BL mPFC) resulted in significant reductions in both alcohol craving and consumption. Similarly, Rapinesi *et al*. [42] used 20 sessions of 18 Hz rTMS (1980 pulses/session) over bilateral DLPFC and found improvements in craving and depressive symptoms.

These discrepancies underscore the influence of factors such as stimulation site, laterality, frequency, and cumulative dose on therapeutic outcomes. Negative findings are frequently associated with insufficient session numbers or stimulation of brain regions less clearly implicated in craving modulation [39, 43]. Moreover, inter-individual differences in neurobiological functioning, including fronto-striatal dysregulation or co-occurring affective symptoms, may further mediate treatment responsiveness.

In contrast, several single-session studies have focused instead on exploring neurobiological mechanisms rather than sustained clinical outcomes. For example, Hanlon *et al*. [44] and Kearney-Ramos *et al*. [45] applied continuous theta burst stimulation (cTBS) to the ventromedial prefrontal cortex (vmPFC) in single session (3600 pulses) and observed no significant reductions in craving. Although these single-session interventions do not yield meaningful clinical effects, they are valuable for delineating the neural circuitry involved and informing the development of more targeted protocols.

In conclusion, multi-session RCTs—particularly those delivering more than ten sessions, using high-frequency stimulation, and targeting right or bilateral DLPFC/mPFC—offer the most compelling evidence for the efficacy of rTMS in reducing alcohol craving [10, 41]. Conversely, single-session studies, though useful for neurobiological insight, generally lack the intensity and duration needed for clinical benefit. Future research should prioritize the optimization of stimulation parameters, refinement of individualized targeting strategies, and incorporation of biomarkers to better predict and monitor treatment response.

## 4. rTMS in the Treatment of Alcohol Use Disorder: Targeting the DLPFC 

Among the most extensively investigated cortical targets for rTMS in AUD is the DLPFC. This region is pivotal for higher-order executive functions, including inhibitory control, working memory, decision-making, and emotion regulation, all of which are commonly impaired in AUD patients [46, 47]. DLPFC dysfunction contributes to deficits in cognitive control, leading to difficulties in resisting alcohol cravings and controlling alcohol consumption, especially in response to cues or stressful situations [23].

The therapeutic potential of DLPFC-targeted rTMS has been evaluated in both single-session and multi-session study designs. For instance, Mishra *et al*. [10]. conducted a multi-session randomized controlled trial (RCT) where high-frequency rTMS (10 Hz) was applied to the right DLPFC over 10 sessions. The study found a significant reduction in alcohol cravings in the active rTMS group compared to the sham group, suggesting the efficacy of multi-session rTMS protocols. Similarly, other multi-session trials have reported that high-frequency rTMS applied to the DLPFC can not only reduced alcohol cravings but also improved executive function, suggesting that bilateral stimulation may produce more pronounced therapeutic effects [48, 49].

Subsequent research has supported the efficacy of rTMS in modulating activity in the DLPFC to reduce alcohol cravings and improve cognitive control in AUD patients. For instance, previous studies demonstrating that high-frequency rTMS applied to the DLPFC not only reduced alcohol cravings but also improved executive functioning, suggesting that bilateral stimulation may produce more pronounced therapeutic effects [48, 49]. These results highlight the potential for rTMS to restore cognitive control and diminish the compulsive behaviors associated with alcohol dependence by normalizing activity within the frontal-striatal circuits involved in addiction.

However, findings across studies remain mixed. For example, Herremans *et al*. [39]. reported that short-term rTMS treatment failed to produce significant reductions in alcohol cravings, suggesting that the duration and frequency of treatment sessions may play a critical role in determining efficacy. Similarly, Höppner *et al*. [38] found no significant changes in craving or alcohol use behavior following high-frequency rTMS applied to the left DLPFC, raising questions about whether unilateral or bilateral stimulation, as well as the specific stimulation parameters, may influence the outcomes of rTMS treatment for AUD. These discrepancies underscore the importance of further research to refine rTMS protocols, optimize stimulation parameters, and identify patient characteristics that may predict treatment response.

The role of placebo response must also be considered. Double-blind, sham-controlled trials indicate that participants often improve regardless of active stimulation, highlighting the need for rigorous placebo control [50]. A sham-controlled study in AUD reported no significant advantages of active high-frequency rTMS over sham for reducing drinking or craving outcomes [50]. For example, a multi-center double-blind trial in substance dependence found that both real and sham rTMS produced comparable reductions in drug craving and use, with no main effect of treatment observed [51]. Therefore, future studies must prioritise stringent sham control and blinding to ensure the observed effects can be confidently attributed to neuromodulation.

Stimulation laterality is another factor potentially moderating outcomes. While the right DLPFC has been frequently targeted with promising results [10], some studies have explored the efficacy of left-sided or bilateral stimulation [41, 52]. Bilateral DLPFC stimulation may yield more pronounced effects by engaging broader networks involved in executive function and craving regulation [42]. Nonetheless, results remain inconsistent, and further investigation is required to delineate the optimal stimulation configuration.

In summary, high-frequency rTMS targeting the DLPFC has shown considerable promise in mitigating alcohol craving and consumption in AUD, particularly when administered via extended, multi-session protocols [10]. Yet, inconsistencies across studies [10, 39, 49, 53]—attributable to variations in stimulation parameters, treatment duration, and sample size—highlight the need for larger, standardized trials. Crucially, long-term durability of rTMS effects remains poorly understood. Continued research is essential to refine DLPFC-targeted protocols and establish their efficacy as a standard intervention for AUD.

## 5. rTMS Targeting the mPFC in AUD Treatment 

Beyond the DLPFC, the mPFC has also emerged as a critical target for rTMS in the treatment of AUD. The mPFC is a central hub for reward processing, decision-making, and emotional regulation, and it plays a pivotal role in modulating the craving and reward circuits that underlie addictive behaviors [37]. Dysregulation of the mPFC, particularly in its connectivity with the ventral striatum (VS), has been implicated in the cravings and relapse that characterize AUD [54]. Study indicates that the mPFC is hyperactive in response to alcohol-related cues in AUD patients, leading to increased craving and relapse susceptibility [55].

Initial investigations into rTMS targeting the mPFC focused primarily on single-session or exploratory pilot study. For example, De Ridder *et al*. [28] conducted a single-session trial using low-frequency (1 Hz) rTMS over the mPFC in a small sample of AUD patients. The study reported a significant short-term reduction in self-reported cravings. While encouraging, the study’s limited scope and absence of long-term follow-up limit its clinical applicability.

Subsequent research has increasingly turned to multi-session RCTs. A notable example is the study by Ceccanti *et al*. [41], which utilized deep rTMS targeting the dorsal mPFC. Participants underwent ten sessions of high-frequency (20 Hz) stimulation across two weeks. The active rTMS group experienced significant reductions in both craving and alcohol intake, with effects sustained at a one-month follow-up, unlike the sham-treated group. These results suggest that repeated stimulation of the mPFC may yield sustained clinical benefits through modulation of craving-related circuitry.

However, not all findings have been consistent. Hanlon *et al*. [44] and Kearney-Ramos *et al*. [45] employed continuous theta burst stimulation (cTBS), a patterned low-frequency rTMS protocol targeting the mPFC, in a single-session trial to investigate its effect on craving in AUD patients. Their study found no significant reduction in craving after a single cTBS session. The authors emphasized that single-session interventions may be insufficient to induce clinically meaningful changes and that longer protocols may be required to observe therapeutic effects.

Variability in outcomes across mPFC-focused studies may be due to multiple factors, including stimulation parameters (e.g., frequency, intensity, and number of sessions), anatomical subregion targeted (e.g., dorsal versus ventral mPFC), and individual patient characteristics (e.g., baseline craving severity, co-occurring psychiatric conditions). The type of rTMS protocol also plays a role: traditional high-frequency stimulation and theta burst stimulation (TBS) may exert differing effects on cortical excitability.

A recent study has explored TBS, a condensed and efficient form of rTMS, as a means of targeting the mPFC. TBS includes intermittent TBS (iTBS), which mimics high-frequency stimulation and is generally excitatory, and continuous TBS (cTBS), which is inhibitory. Hanlon *et al*.’s work [44] on cTBS showed that while a single session had minimal behavioral effect, repeated application might produce cumulative changes in craving-related circuits.

In summary, rTMS targeting the mPFC demonstrates potential for reducing alcohol craving in AUD, supported by both mechanistic insights and emerging clinical evidence. Single-session studies suggest transient modulation of craving-related circuits, while multi-session protocols—especially those using deep or bilateral stimulation—have yielded more consistent reductions in craving and drinking behavior [28, 41]. Further research is needed to determine the optimal stimulation parameters, target locations within the mPFC, and whether specific subpopulations of AUD patients (e.g., those with high cue-reactivity or emotion dysregulation) benefit more from mPFC-focused interventions.

## 6. Neuroimaging Studies on rTMS in AUD Treatment: Uncovering Deep Neural Mechanisms 

The application of neuroimaging techniques has greatly advanced our understanding of the neural mechanisms underlying rTMS treatment in AUD. facilitates the characterization of structural and functional brain abnormalities in individuals with AUD but also provides insights into how rTMS modulates these circuits to exert its therapeutic effects. Several neuroimaging modalities, including functional magnetic resonance imaging (fMRI), and Single Photon Emission Computed Tomography (SPECT) [56], have been employed to assess the effects of rTMS on brain activity and connectivity in AUD patients.

One prominent finding is that rTMS targeting the DLPFC acutely alters activity in subcortical regions such as the striatum [57] and thalamus, which are critical for reward-driven behavior and habit formation [58]. These results support the hypothesis that rTMS may restore the balance of activity between the cognitive control and reward circuits in AUD, thereby reducing cravings and improving self-regulation over alcohol consumption. However, it is important to note that these conclusions are primarily derived from short-term, single-session neuroimaging studies, with a paucity of longitudinal clinical imaging data.

A notable exception is the study by Jansen *et al*. [53] who used fMRI to assess changes in brain connectivity following 10 sessions of high-frequency rTMS applied to the right DLPFC in patients with AUD. The results demonstrated enhanced resting-state functional connectivity within the frontoparietal network—a circuit associated with executive control—a post-intervention. These findings suggest that repeated rTMS sessions may strengthen the functional integration of cognitive control networks, potentially improving craving regulation and reducing relapse risk.

In a SPECT imaging study, Addolorato *et al*. [56] explored the effects of multi-session deep rTMS on dopamine transporter (DAT) availability in the striatum. They reported that deep rTMS significantly reduced DAT binding, interpreted as a sign of increased dopaminergic tone. Given the central role of dopamine in reinforcement and addiction, such changes may underlie some of the behavioral improvements observed in rTMS-treated AUD patients.

Collectively, these studies illustrate two primary roles of neuroimaging in rTMS research on AUD. First, single-session experiments are instrumental in revealing short-term neural mechanisms—such as changes in connectivity or neurotransmitter dynamics—that may precede behavioral change. Second, multi-session imaging studies begin to shed light on more durable network reorganization and neuromodulatory adaptations following repeated rTMS interventions.

Despite these advances, the neuroimaging literature remains limited by small sample sizes, variability in study protocols, and a lack of extended follow-up. Furthermore, most existing research focuses on resting-state imaging; comparatively few studies utilize task-based fMRI or integrate multimodal approaches such as magnetic resonance spectroscopy (MRS) or diffusion tensor imaging (DTI) to capture the broader impact of rTMS on brain structure and function.

In summary, neuroimaging has significantly enhanced our understanding of how rTMS modulates the neural circuitry implicated in AUD. Nevertheless, there is a clear need for larger, longitudinal, and multimodal neuroimaging investigations. Future work should aim to identify neurobiological predictors of clinical response, characterize how these neural changes unfold over time, and evaluate the potential for neuroimaging to inform the development of individualized rTMS treatment protocols in AUD.

## 7. Future Directions in rTMS Research for AUD Treatment 

Despite the growing body of evidence supporting the therapeutic potential of rTMS in AUD, several critical challenges remain unresolved. key issue is the lack of consensus regarding optimal stimulation parameters. While both high- and low-frequency rTMS protocols have demonstrated efficacy, the most effective combination of stimulation frequency, intensity, and session duration for producing robust and sustained clinical outcomes remains unclear [43, 59]. Furthermore, inter-individual variability in neurobiological and psychological profiles may substantially responsiveness to treatment responsiveness, highlighting the need for personalized rTMS protocols.

Personalization strategies may involve the use of neuroimaging modalities such as fMRI or SPECT to identify dysregulated neural circuits in AUD patients and guide stimulation accordingly. For instance, individuals exhibiting hyperactivity in the ventral striatum (VS) and mPFC may benefit from rTMS targeting these areas to attenuate craving-related neural activity [54, 55]. Conversely, patients with impaired cognitive control resulting from hypoactivity in the DLPFC may derive greater benefit from stimulation of this region to enhance executive function and reduce susceptibility to alcohol-related cues [23].

To address variability and enhance reproducibility, future research should adopt standardized rTMS protocols (e.g., consistent target site, frequency, and number of sessions), and investigate maintenance strategies to sustain effects. Long-term follow-ups are needed to assess treatment durability. Additionally, studies should explore personalized approaches, such as using neuroimaging to tailor stimulation targets to individual brain circuitry. These strategies align with recent recommendations to optimize neuromodulation interventions and would improve understanding of rTMS mechanisms and outcomes by accounting for individual variability.

In addition to protocol refinement, integrating rTMS with other therapeutic modalities offers a promising strategy for enhancing treatment efficacy. Pharmacological agents such as naltrexone or acamprosate may complement the neuromodulatory effects of rTMS by targeting distinct biochemical pathways. Likewise, behavioral interventions such as cognitive-behavioral therapy (CBT) or mindfulness-based therapies may synergistically reinforce cognitive control and reduce relapse risk [21, 22]. An integrated treatment framework could yield more comprehensive and durable benefits for individuals with AUD.

An additional promising direction involves the exploration of advanced stimulation paradigms, particularly theta burst stimulation (TBS). TBS protocols, which include intermittent TBS (iTBS) and continuous TBS (cTBS), have shown potential to induce neuroplastic effects equivalent to or greater than conventional rTMS, while significantly reducing treatment time [30]. Preliminary studies suggest that TBS may offer comparable anti-craving and cognitive benefits in AUD and other substance use disorders. However, findings have been inconsistent, and further investigation is warranted to determine whether TBS provides superior clinical outcomes and greater efficiency relative to standard protocols.

Finally, long-term effectiveness remains a key concern. While short-term studies indicate that rTMS can reduce alcohol craving and enhance executive function, it remains unclear whether these effects are sustained over extended periods. Well-designed longitudinal trials are essential to determine whether rTMS produces lasting neural and behavioral changes that contribute to relapse prevention and support long-term recovery in patients with AUD.

## 8. Applications of rTMS in Other Substance and Behavioral Addictions

Beyond AUD, rTMS has been applied to other substance use disorders (SUDs)—including nicotine, cocaine, and cannabis—as well as behavioral addictions like gambling and internet gaming. These conditions share underlying neurocircuitry disruptions, particularly in fronto-striatal pathways governing reward and self-control [60].

**Nicotine addiction**: In tobacco use disorder, high-frequency rTMS targeting the left DLPFC can acutely decrease cigarette craving, and several sham-controlled trials have demonstrated reductions in smoking consumption [61]. For example, five daily sessions of left DLPFC rTMS significantly dampened nicotine craving while increasing activity in frontal cognitive-control regions and striatum, and strengthening connectivity between these executive and reward networks [24]. These neural changes suggest that rTMS helps restore top-down inhibitory control over drug-seeking [24].

**Stimulant and other SUDs**: In cocaine and other stimulant addictions, repeated high-frequency rTMS over the DLPFC has been shown to reliably reduce drug craving, and one study has further reported a decrease in substance use following treatment [62]. Both standard 15 Hz protocols and accelerated theta-burst rTMS have yielded comparable anti-craving effects in cocaine use disorder, indicating flexibility in neuromodulation approaches [63]. Preliminary trials in cannabis use disorder similarly suggest that rTMS can decrease cannabis intake [64], though this work remains in early stages.

**Behavioral addictions**: Although research is nascent, rTMS has shown promising results in behavioral addictions. Gambling disorder—which involves fronto-striatal dysregulation similar to SUDs [62]—appears responsive to prefrontal neuromodulation. In a recent trial, two weeks of high-frequency left DLPFC rTMS induced a robust decrease in gambling craving that persisted up to 24 weeks, whereas the sham-treated group’s cravings returned to baseline by 12 weeks [65]. Likewise, case reports in internet gaming disorder and compulsive online pornography use noted reduced addictive behaviors and improved executive control following DLPFC-rTMS [60].

These findings support the broader application of rTMS beyond AUD. However, more research is needed to determine optimal protocols, target sites, and individual predictors of response. Future directions include integrating rTMS with behavioral therapies, using neuroimaging to guide treatment personalization, and conducting large-scale trials in underexplored addiction subtypes.

## 9. Conclusion

rTMS represents a promising therapeutic approach for the treatment of AUD, offering a non-invasive method for modulating the neural circuits involved in craving regulation and cognitive control. By targeting key cortical regions such as the DLPFC and mPFC, rTMS has been shown to attenuate alcohol cravings, enhance executive function, and induce long-term neuroplastic changes in the brain. Neuroimaging studies have provided valuable insights into the mechanisms by which rTMS exerts its therapeutic effects, particularly its ability to modulate activity in frontal-striatal circuits and dopaminergic pathways.

Despite these advances, several critical challenges persist. Further research is needed to refine stimulation protocols, including the optimal frequency, intensity, and session duration for diverse patient populations. Personalized approaches based on individual neurobiological profiles—potentially guided by neuroimaging biomarkers—may enhance therapeutic efficacy and minimize inter-individual variability in treatment response. In addition, the long-term durability of rTMS effects on relapse prevention remains inadequately understood and warrants longitudinal investigation. With continued methodological refinement and empirical validation, rTMS has the potential to become an integral component of evidence-based treatment strategies for alcohol dependence, addressing a critical gap in current clinical practice.
